# Editorial: The Search for Biological Active Agent(s) From Actinobacteria

**DOI:** 10.3389/fmicb.2018.00824

**Published:** 2018-05-01

**Authors:** Learn-Han Lee, Kok-Gan Chan, Jem Stach, Elizabeth M. H. Wellington, Bey-Hing Goh

**Affiliations:** ^1^Novel Bacteria and Drug Discovery Research Group, School of Pharmacy, Monash University Malaysia, Bandar Sunway, Malaysia; ^2^Biofunctional Molecule Exploratory Research Group, School of Pharmacy, Monash University Malaysia, Bandar Sunway, Malaysia; ^3^Biomedical Research Laboratory, Jeffrey Cheah School of Medicine and Health Sciences, Monash University Malaysia, Bandar Sunway, Malaysia; ^4^Center of Health Outcomes Research and Therapeutic Safety, School of Pharmaceutical Sciences, University of Phayao, Phayao, Thailand; ^5^International Genome Centre, Jiangsu University, Zhenjiang, China; ^6^Division of Genetics and Molecular Biology, Institute of Biological Sciences, Faculty of Science, University of Malaya, Kuala Lumpur, Malaysia; ^7^School of Natural & Environment Sciences, Newcastle University, Newcastle Upon Tyne, United Kingdom; ^8^School of Life Sciences, University of Warwick, Coventry, United Kingdom

**Keywords:** actinobacteria, genome, *Streptomyces*, soil, marine, bioactive

## Introduction

Nature has always been an interesting source for bioactive products, particularly those derived from microorganisms. Even though microorganisms can be found literary throughout Earth, more efforts are still needed to study the microbial biodiversity, given that there are still 99.999% of microbial taxa that awaits to be discovered (Locey and Lennon, 2016). As the largest phylum under the Bacteria kingdom, *Actinobacteria* has gained tremendous amount of attention from the scientific community, mainly due to their ability in producing a vast array of bioactive compounds with interesting chemical structures (Barka et al., 2016). To date, actinobacteria have contributed more than 65% of antibiotics used in medicine; out of which over 10,000 bioactive compounds were produced by the members of the genus *Streptomyces* (Bérdy, 2005; Bull and Stach, 2007; Subramani and Aalbersberg, 2012; Zotchev, 2012; Karuppiah et al., 2016). Even after decades of bioprospecting research, the genus *Streptomyces* remains in the spotlight of microbial product research, mainly due to its seemingly unsurpassed ability in synthesizing a vast array of compounds with various bioactivities (Forget et al.; Kamjam et al.; Law et al.; Lyu et al.; Maryam and Khan; Tan et al., 2016; Ser et al., 2017). The genus *Streptomyces* was firstly introduced by Waksman and Henrici in 1943, several years before Professor Waksman was bestowed with the Nobel Prize in Physiology or Medicine in 1952 for the discovery of streptomycin from *Streptomyces griseus* (Schatz et al., 1944; Nobelprize.org., 2018). Since then, continuous efforts have been put in to explore the potential of these Gram-positive bacteria and the genus now contains over 860 species and subspecies isolated from various habitats (www.bacterio.net). One of the reasons behind their ubiquitous nature is the unique developmental life cycle—these bacteria grow to form substrate mycelium and further develop spores when the environment becomes unfavorable for growth. Facing this environmental stress, some actinobacteria have also deployed defense system and/or survival mechanism like production of secondary metabolites that may have more functions than we initially thought.

Under the research topic “The search for biological active agent(s) from actinobacteria,” a total of 23 articles were published, covering a variety of topics revolving actinobacteria including their diversity in different habitats and the discovery of novel, bioactive strains along with some interesting bioactivities such as antibacterial, antifungal, antioxidant, and anticancer properties.

Due to the ease of sampling, numerous literature has previously indicated diversity of terrestrial-derived actinobacteria across the globe. For instance, Professor William C. Campbell and Professor Satoshi Omura have successfully discovered *Streptomyces avermitilis* (from golf course in Ito) which synthesized an antiparasitic compound, avermectin, and subsequently earn them (part of) the Nobel Prize of Physiology and Medicine in 2015 (Burg et al., 1979; Nobelprize.org., 2018). Even after extensive search of bioactive strains from terrestrial area, there are some actinobacteria hidden in parts of world/geographical region that have been previously overlooked (Sharma et al.; Khieu et al., 2015). For example, one of the study has discovered rich diversity of antimicrobial producing *Streptomyces* from moonmilk cave, which is locally used as traditional medicine against several ailments (Maciejewska et al.). The same study reported that 90% of the strains isolated from the cave induced strong growth suppression against the multi-drug resistant *Rasamsonia argillacea*, thus possessed great potential to be developed for cystic fibrosis patients and those with chronic granulomatous diseases. On the other hand, researchers have also reported presence of bioactive *Streptomyces* sp. agricultural soil in Beni-Suef, Egypt and successfully discovered a diketopiperazine derivative (m/z 488.05) which may contribute to antimicrobial and anticancer activities (Ahmad et al.). On the other hand, *Streptomyces* sp. ASK2 was shown to produce an aromatic compound with aliphatic side chain (m/z 444.43) exhibiting antagonistic activity against multidrug resistant *K. pneumoniae* using adult zebrafish infection model (Cheepurupalli et al.). Furthermore, actinobacteria found in rhizosphere soil of plants and endophytic actinobacteria (e.g., reside on/within certain plant species) contribute as good source of bioactive compounds, as they could synthesize a wide diversity of secondary metabolites which may promote and/or ensure health of their host. Growing in the wetland area, 10 actinobacteria strains were recovered from the medicinal plant *Vochysia divergens* located in wetland area in Brazil, belonging to the *Aeromicrobium, Actinomadura, Microbacterium, Microbispora, Micrococcus, Sphaerisporangium, Streptomyces*, and *Williamsia* genera (Gos et al.). One of the extract produced by strain LGMB491 (a close relative of *Aeromicrobium ponti*) displayed the highest activity against methicillin-resistant *Staphylococcus aureus* (MRSA), with a minimum inhibitory concentration of 0.04 mg/mL. Strain LGMB491's extract contained 1-acetyl-β-carboline (1), indole-3-carbaldehyde (2), 3-(hydroxyacetyl)-indole (4), brevianamide F (5), and cyclo-(L-Pro-L-Phe) (6) as major compounds with antibacterial activity. Though, more than 50 years have passed since the discovery of streptomycin from soil streptomycete, these papers have once again demonstrated the pharmaceutical importance of terrestrial-derived actinobacteria.

Actinobacteria are abundance in nature, therefore some studies have expanded from the initial isolation source (i.e., terrestrial area) and began to search for actinobacteria from freshwater and marine environments. As over 70% of the Earth is covered by water (e.g., lake, sea, ocean), it is almost close to impossible not to encounter any actinobacteria with unique traits (Balasubramanian et al.; Dhakal et al.; Islam et al.; Quezada et al.; Undabarrena et al.). As discussed by Kamjam et al., there have been more than 21 new species of 13 genera reported between year 2006 and 2016. Some of which could produce secondary metabolites, among which *Streptomyces* species is the richest producer. By the same token, marine soil/sediments have also demonstrated similar bioactive potential as those derived from terrestrial sources; several actinobacteria strains isolated in India displayed strong antimicrobial activity against Gram-positive bacteria, Gram-negative bacteria, and *Candida albicans* (Dholakiya et al.; Kavitha and Savithri). Furthermore, bioprospecting of actinobacteria from dynamic environment such as the mangrove areas are gaining good results as well. Many studies were from Asia, an area that has the largest coverage of mangrove forests which equates to 42% of the total global mangrove area (Giri et al., 2011; ITTO, 2014). Malaysia is home to 3.7% of global total mangrove coverage and three studies in this research topic have emphasized the importance of this ecosystem, particularly for the search of bioactive compounds from actinobacteria (Arumugam et al., 2013; Lee et al., 2014; Ser et al., 2015, 2016; Azman et al., 2017). The hexane partition of *Streptomyces* sp. CCB-PSK207 was found to be able to protect *Caenorhabditis elegans* against with *Pseudomonas aeruginosa* strain PA14 infection via re-activation of lysozyme 7, without impairing feeding behavior of *C. elegans* (Fatin et al.). These findings have revealed a key component for *P. aeruginosa* PA14 infection—lysozyme 7 which functions as innate immunity defense molecule and this was the first report of marine actinobacteria producing metabolites which is capable of rescuing *C. elegans* from PA14 through modulation of lys-7 activity. Another two novel *Streptomyces* species have also been isolated from different parts of Malaysia: (a) *Streptomyces colonosanans* MUSC 93J^T^ from Sarawak, East Malaysia, and (b) *Streptomyces antioxidans* MUSC 164^T^ from east coast of Peninsular Malaysia. MUSC 93J^T^ was given the name as *S. colonosanans* given that the strain demonstrated anticancer activity against human colon cancer cell lines without significant cytotoxic effect against human normal colon cells (Law et al.). On the other hand, *S. antioxidans* MUSC 164^T^ was found to produce pyrazines and phenolic-related compounds which are capable of reducing free radicals and protect neurons against hydrogen peroxide damages (Ser et al.). Altogether, these studies suggested the importance of aquatic associated actinobacteria, especially against harmful pathogens and chronic human diseases like neurodegenerative diseases and cancer.

Exploring new taxa has always been successful strategy in the discovery of candidates for the development of new microbial drug(s). However, the subsequent steps to maximize yield and production from the species of interest are equally important. As part of the commonly used antibiotics, clavulanic acid is initially isolated from *Streptomyces clavuligerus*. Ser et al. discussed the importance of optimization in traditional fermentation technology, particularly fermentation conditions and media composition to increase clavulanic acid production in *S. clavuligerus*. In fact, the differences in major ingredient(s) of a medium is imperative as slight changes in composition (or concentration) could tip off the balance between the growth of the organism and the production of secondary metabolite(s). Using clavulanic acid as example, the addition of its precursors like arginine and ornithine increase the supply of C5 precursors into the biosynthesis pathway, which ultimately lead to higher production of the antibiotics. Similarly, one could also increase the yield of compound of interest by reducing occurrence of competing pathway, either via addition of specific inhibitors or even reprogram metabolic pathways via genetic modifications (Pickens et al., 2011).

With the advancement in next-generation sequencing (NGS), actinobacteria are recognized as “hidden treasures” in the nature that awaits exploration (Jose and Jha). In point of fact, the availability of genome sequences has unlocked possibly new potential of actinobacteria (van Heel et al., 2013; Skinnider et al., 2015; Blin et al., 2017; Ser et al., 2018). Following stimulated much progress in computational resources, including bioinformatics tools like antibiotics and Secondary Metabolites Analysis SHell (antiSMASH) and Prediction Informatics for Secondary Metabolomes (PRISM) to assist in surveying of the genomic the announcement of the complete genome sequence for the model actinobacterium *Streptomyces coelicolor* A3(2), many researchers have discovered presence of “cryptic” or silent biosynthetic gene clusters among actinobacteria genomes, indicating that researchers may have underestimated the “true nature” of actinobacteria specifically in producing useful secondary metabolites (Bentley et al., 2002; Takagi and Shin-ya, 2011; Harrison and Studholme, 2014). As a result, several studies have employed the CRISPR/Cas9 system to increase production yield, by (a) either knock-in genes to activate silent biosynthetic gene clusters or (b) delete repressors genes (Pickens et al., 2011; Jia et al., 2017; Robertsen et al., 2017; Zhang et al., 2017). By introducing a heterologous promoter, kasO^*^ promoter in the upstream region of the biosynthetic gene clusters, Zhang et al. (2017) have successfully “awaken” the silent biosynthetic gene cluster in *Streptomyces roseosporus* NRRL15998 and stimulated production of two polycyclic tetramate macrolactam, which failed to be expressed in a heterologous system. Altogether, metabolic engineering offers an alternative to shorten time needed to optimize a strain, possibly simplifying the subsequent purification process to isolate compound of interest. Coupled with the cutting-edge molecular and analytical tools, the discovery of biosynthetic gene clusters could possibly increase the chemical diversity of actinobacteria from different natural sources, consequently amplifying the pharmaceutical potential of these beneficial microbes.

## Author contributions

All authors listed have made substantial, direct and intellectual contribution to the work, and approved it for publication.

### Conflict of interest statement

The authors declare that the research was conducted in the absence of any commercial or financial relationships that could be construed as a potential conflict of interest.
